# Suffering and happiness in Turkish folk poetry in the context of positive psychology: The examples of Asik Mahzuni Serif and Neset Ertas

**DOI:** 10.3389/fpsyg.2022.1104611

**Published:** 2023-01-17

**Authors:** Ahmet Zeki Güven, Gökmen Arslan

**Affiliations:** ^1^Faculty of Education, Department of Turkish Language Education, Akdeniz University, Antalya, Turkey; ^2^Department of Psychological Counseling and Guidance, Mehmet Akif Ersoy University, Burdur, Turkey; ^3^Centre for Wellbeing Science, University of Melbourne, Australia

**Keywords:** positive psychology, Turkish folk poetry, suffering, happiness, cultural communication

## Abstract

The widespread adoption of positive psychology at the beginning of the century has fortified the scholarly foundations of “happiness.” Thus, researchers have focused on “happiness” rather than “suffering” in boosting the joy of life within positive psychology, aiming for individuals to achieve peace with themselves and society. With the developments in positive psychology, over recent years, the idea of integrating both positive and negative aspects of human nature to build a better life for oneself and others has contributed to the rise of second-wave positive psychology (PP 2.0). The present study aimed to explore suffering and happiness in Turkish folk culture through a sample of poems by *Asik Mahzuni Serif* and *Neset Ertas*. The study results indicated that suffering-themed concepts were mentioned more than happiness-themed concepts. Within the theme of suffering, the world was the most frequently mentioned concept in Mahzuni’s works. He emphasizes in his works that the world is the source of many sufferings. In Ertas’s poems, moreover, love was found to be the most frequently mentioned suffering-themed concept. Ertas considers love to be the most significant source of suffering. It was also determined that while separation is the least used concept in the theme of suffering in Mahzuni’s verses, it is never mentioned in Ertas’s poems. Other concepts pointing to the theme of suffering are poverty, ignorance, longing, death, and slavery. We found that the theme of happiness is mentioned much less frequently than the theme of suffering. While the most used happiness-themed concept is misery/remedy, in Mahzuni’s words, love is cited in Ertas’s poems. Expressing the view that suffering can be an opportunity for people, Mahzuni emphasizes in his poems that people can grow by learning lessons from their suffering. Ertas, moreover, sees love as the most important source of happiness. The other concepts referencing happiness in the poems were friend, mother, soft answer, and spring. Overall, the results suggest that suffering is an important source of building resilience, which, in turn, can produce happiness. People can grow with the help of the experience of suffering so that this experience can contribute to their flourishing.

## 1. Introduction

The concept of positive psychology, which centers on positive character traits, strengths, and virtues and aims to contribute to the individual’s attainment of happiness, is not new. The notion that people may improve their flourishing has persisted through the centuries and across cultures. Thus, the background to the concept dates much further back. Throughout the ages, philosophers have always attempted to reveal the significance of a decent, virtuous, and moral life. For example, Aristotle, the famous Greek philosopher, also drew attention to the importance of noble life and emphasized happiness as the highest good for humanity (Taylor, 2001). Maslow (1954) argued that psychology was a branch of science that had been successful in focusing on the adverse aspects of personality traits; however, it seemed to ignore the individual’s strengths and potential. Approximately 40 years after Maslow emphasized the importance of an individual’s strengths and potential, Seligman asserted that psychology should be more involved in the positive aspects of human nature (Lopez and Gallagher, 2011). Seligman argued that psychology had ignored its ultimate missions, such as curing mental disorders, identifying individuals’ abilities, and contributing to their enjoyment of a more productive and meaningful life (Linley, 2009). Seligman then called on psychologists to create a science of character and virtue that would nurture the best in people. Shortly after this call, he announced the development of positive psychology to offer people a robust vision of life. According to Seligman and Csikszentmihalyi (2000), the fundamental task of psychology is not only to fix an individual’s undesirable states but also to work on their strengths and virtues (Sheldon and King, 2001).

Positive psychology is also the scientific study of optimal human functioning with the goal of understanding the factors and states that enable people and communities to thrive (Seligman and Csikszentmihalyi, 2000). From this perspective, many variables (e.g., optimism, hope, gratitude, social support, humor, creativity, forgiveness, and self-confidence) fall within the scope of positive psychology (Lopez and Gallagher, 2011). In particular, the widespread adoption of positive psychology at the beginning of the century has fortified the scholarly foundations of “happiness.” Thus, researchers have widely focused on “happiness” rather than “suffering” in boosting the joy of life within positive psychology, aiming for individuals to achieve peace with themselves and society. With the recent developments in positive psychology, the idea of integrating both positive and negative aspects of human nature to build a better life for oneself and others has contributed to the rise of second-wave positive psychology (PP 2.0; Wong, 2010, 2019). It not only focuses on life’s positive aspects but also welcomes its adverse aspects as a whole. In addition, focusing on both positive and negative psychological qualities and states will contribute more to the wellbeing of the individual and society, and such a focus should be a seminal research area of psychology (Lomas, 2016; Arslan, 2019; Yıldırım, 2019; Burke and Arslan, 2021; Arslan and Wong, 2022). As such, second-wave positive psychology (PP 2.0) emphasizes that we may need to accept that life have negative aspects, as well as positive ones (Wong, 2010; Lomas, 2016).

### 1.1. Suffering and happiness in Turkish folk poetry in a cultural context

The literature features a plethora of studies on positive psychology that point out the importance of happiness (Diener, 1984; Lyubomirsky, 2007). Diener (1984) defines happiness as experiencing frequent and intense emotions and life satisfaction. According to Michalos (2008), a happy person is one who demonstrates less fear, hatred, tension, guilt, and anger but more energy and vitality; who is self-confident and emotionally stable, healthy, and fulfilling; who has a high social orientation, love, social relationships, an active lifestyle, and a meaningful job; and who is relatively optimistic, carefree, and present-oriented. While positive psychology is widely about happiness and emotions, Wong’s PP 2.0 (Wong, 2019) has focused on suffering as the gateway to happiness and argued that happiness is impossible without overcoming the dark side of human life (Wong, 2020). Therefore, second-wave positive psychology stresses the assumptions that suffering is necessary for happiness, and enduring happiness or flourishing can only be achieved through the dialectical integration of opposites (Wong et al., 2021).

The meanings attributed to “suffering” and “happiness” may vary by culture, and PP 2.0 emphasizes the importance of understanding the unique experience and expression of happiness in different cultures (Wong, 2011). For example, some cultures enjoy high levels of happiness despite being fraught with crime and economic difficulties (Veenhoven, 2022), which may be because individuals adopt values, beliefs, feelings, thoughts, standards, schemas, and judgments for suffering or happiness through their cultures. In this sense, the analysis of culture may bring noteworthy contributions to explaining “happiness” and evaluating individuals’ behaviors and personal characteristics. The impact of culture on individuals is often visible through values. Folk culture, an important indicator of culture, has an important function in maintaining social life through creating, nurturing, and transferring social values, judgments, beliefs, and thoughts to other generations. More specifically, folk poetry and songs in folk culture are more evident reflections of culture; thus, Turkish folk poetry embodies significant traces of Turkish culture. Folk poets often address society’s cultural characteristics in their verses; their poems sometimes express suffering and sometimes convey happiness. In these poems, one may encounter deteriorations in the social structure due to neglect of science or elevated injustice. Folk poetry functions as a mirror of both culture and society, allowing us to understand the social and cultural structures of a specific period. This is also the case in Turkish folk poetry; Turkish folk poets sometimes talk about happiness with love and sometimes suffering due to longing for loved ones.

### 1.2. Purpose of the study

The present study attempted to trace suffering and happiness in Turkish folk poetry. Accordingly, poems in Turkish folk poetry were investigated and interpreted based on the emerging schemas of “suffering” and “happiness” in the context of second-wave positive psychology. This study aimed to provide a further understanding of suffering and happiness in the Turkish folk culture through a sample of poems by Asik Mahzuni Serif and Neset Ertas. A total of 240 poems by Neset Ertas were extracted from *Garip Bulbul Neset Ertas* (Neset Ertas, A Lonely Nightingale; Parlak, 2013), while 144 poems by Asik Mahzuni Serif were taken from the book *Iste Bizim Mahzuni* (Here is Our Mahzuni; Yağız, 1999). However, it should be noted that these poems may not correspond to the total number of works produced throughout the poets’ lives since they must have produced poems that have never been released. Almost all the verses of both poets are composed as folk songs. While the logic of traditional folk songs relies on anonymity, these poems were so popular and well known that nobody thought about their anonymity. In this study, the selected poems were coded and interpreted around suffering and happiness in Turkish culture. Considering that the literature hosts no such comparative study seeking the traces of positive psychology in Turkish folk poetry, it can confidently be asserted that the findings will bring significant contributions to the field.

## 2. Method

The research employed cultural analysis, a qualitative research method. The aim of cultural analysis is to define and interpret the culture of a particular group of people within the concepts, processes, and perceptions pertaining to that culture. Thus, every detail about the target subject needs to be investigated in-depth. The data were collected using the document analysis technique, a data collection technique in qualitative research. In this regard, the researcher first went through the selected poems two times. The researcher then grouped the poems by their themes of suffering and happiness and determined the codes for each theme. This was used to generate the frequency of keywords related to suffering and happiness in the poems. Finally, the researcher interpreted the findings in light of the biographies of both poets and examined the extent to which the poems reflected the general attitudes and opinions of society through the previous findings. An effort was then made to infer cultural-specific implications about suffering and happiness from the themes and codes. Accordingly, the researcher collected 144 poems by Mahzuni and 240 poems by Ertas, determined the codes of the themes mentioned, and attempted to uncover how the poets addressed suffering and happiness in their poems.

### 2.1. Validity and reliability of the study

To contribute to the reliability of the research, the researcher analyzed the data two times at a specific time interval (about 3 months) and considered the compatibility ratio between the themes and codes emerging in both analyses. Accordingly, the specified ratio was calculated to be 0.94 using the formula suggested by Miles and Huberman (2016). Therefore, it can be argued that the analysis performed in this research was highly reliable.

With regard to validity, the researcher considered credibility, transferability, dependability, and confirmability, as proposed by Lincoln and Guba (1986). In terms of credibility, the researcher reviewed the relevant literature and included the relevant findings in section 1. In addition, the problem situation was clearly presented to promote credibility. The research design and methods are explained in detail in section 2 to promote the transferability of the study. In addition, a detailed explanation of the data analysis process is believed to contribute to the transferability. The present study clearly describes the data analysis process and the data obtained through the themes and codes to improve the dependability of the research. Finally, to ensure the confirmability of the research, the researchers collected the published poems by both poets and did not rely on the poets’ life stories and thoughts in their poems to determine the codes and themes.

## 3. Results

In this study, suffering and happiness were sought in Turkish folk poetry in the context of positive psychology, with the researcher analyzing 144 poems by Mahzuni and 240 poems by Ertas. The findings revealed that the poems by both poets mainly addressed concepts that would evoke suffering (f: 400) and that the frequency of expressions evoking happiness was almost one-third of those prompting suffering (f: 139; Table 1).

**Table 1:** Suffering and happiness in the Turkish folk poetry.

	Suffering					Happiness			
Themes	Mahzuni Serif	(%)	Neset Ertas	(%)	Themes	Mahzuni Serif	(%)	Neset Ertas	(%)
World	35	33	75	25.5	Love	2	28.5	89	67.5
Poverty	24	22.5	13	4.4	Friend	-	-	17	12.9
Love	20	18.7	91	31	Mother	-	-	14	10.6
Ignorance	8	7.4	10	3.5	Soft answer	-	-	8	6
Longing	7	6.6	65	22.1	Spring	-	-	4	3
Death	3	2.7	10	3.4	Misery/remedy	5	71.5	-	-
Slavery	5	4.6	9	3					
Injustice	4	3.6	-	-					
Separation	-	-	21	7.1					
Sub-total	106		294			7		132	
Total	400					139			

### 3.1. Suffering in the poems of Mahzuni and Ertas

The results revealed that nine suffering-related concepts were used 400 times in total, 106 times by Mahzuni and 294 times by Ertas, on the theme of suffering. The identified concepts are presented later.

#### 3.1.1. The world

In the poems analyzed, the world is considered a temporary place where people cannot be happy and are always worried. Accordingly, the world order is characterized by suffering. In fact, the world is such a place that only liars and bad people would be happy. Mahzuni and Ertas emphasize that the world is not a place of happiness but suffering.

Mahzuni uses “world” the most as a concept to evoke suffering (f: 35). According to him, it is a world of liars that hurts people. It is almost a cemetery and does not make him excited. He has never been happy in the world:

*What they call the world is a cemetery*

*I do not know when it makes me happy*

*I’m a flower blooming in the vineyard of friendship*

*The wind of the ignorant makes me wither*

(Dünya dedikleri mezarlık imiş

Bilmem ki ne zaman güldürür beni

Bir çiçeğim verdim dostluk bağında

Eser cahilin rüzgarı soldurur beni; Yağız, 1999, p. 99).

 

*Do not disturb transitory life (world).*

*It cozily hangs out.*

*My tears are hidden to me*

*It goes watering the troubled and the carefree*

(Dokunma keyfine yalan dünyanın

İpini eline dolamış gider

Gözlerim yaşı bana gizlidir

Dertliyi dertsizi sulamış gider; Yağız, 1999, p. 129).

 

*My heart’s mourning never ends*

*What would I do with the world of the cruel?*

*Not as good as an ant*

*Those who lavish advice on me, oh dear!*

(Bir zaman bitmiyor gönlümün yası

Neyleyim ki dünya kahpe dünyası

Bir karınca kadar yoktur faydası

Bol keseden öğüt verenler vah vah; Yağız, 1999, p. 95).

Ertas also uses “world” 75 times in his poems to imply suffering. Like Mahzuni, he states that the world is a lie, a place where people cannot be happy and cannot find love:

*Oh! In this fake world.*

*I wanted to be happy but could not …*

*The love of my strange heart*

*I wanted to find it but could not …*

(Ah şu yalancı dünyada

Gülem dedim, gülemedim

Garip gönlümün yarini

Bulam dedim, bulamadım; Parlak, 2013, p. 313).

This world is a realm in which humans are always aggrieved:

*A lonely man, troubled in the world …*

*Set off upon drinking the poison of the suffering.*

*Unfortunately, he failed to find the way,*

*Confused on the way, leaving …*

(Bir garip, dünyada derdin elinden

Derdin zehrini içmiş, gidiyor.

Ne çare ki, gidememiş yolundan

Kaybetmiş yolunu; şaşmış, gidiyor; Parlak, 2013, p. 350).

For Ertas, the world is temporary and a big lie. We suffer all the time and cannot be happy and laugh in this world.

*Are you the only one bewailing your fate? (x2)*

*I could not be happy either in the fake world*

*Do you think me happy?*

*In the world stealing my life in vain*

*Oh! In the fake world (x2)*

*In the world pretending to smile at my face*

(Hep sen mi yandın, hep sen mi yandın?

Ben de gülemedim yalan dünyada

Sen beni gönlünce mutlu mu sandın?

Ömrümü boş yere çalan dünyada.

Ah yalan dünyada, yalan dünyada.

Yalandan yüzüme gülen dünyada; Parlak, 2013, p. 431).

#### 3.1.2. Poverty

For both poets, poverty is an apparent reason for human suffering since those without sufficient financial means would be in a constant state of deprivation, suffering, and unhappiness.

“Poverty” is mentioned 24 times in Mahzuni’s poems. In one of his poems, he states that some people are unfair to others, and that is why people become poorer:

*Those cheating the poor of their rights never end*

*More than flesh and blood can bear*

*The brave is in need even of onions.*

*I do not know, should I burst it out or not?*

(Yoksulun sırtından doyan doyana.

Bunu gören yürek nasıl dayana.

Yiğit muhtaç olmuş kuru soğana.

ilmem söylesem mi, söylemesem mi?; Yağız, 1999, p. 73).

Uttering that such injustices hurt people, the poet claims that the suffering would lead people to age:

*Mahzuni Serif, kill your pain!*

*Sometimes, find your remedy in misery*

*Like Pir Sultans, the gallows*

*I do not know, should I get hanged on or not?*

(Mahzunî Serif’im dindir acını

Bazan acılardan al ilacını

Pir Sultanlar gibi darağacını

Bilmem boylasam mı, boylamasam mı?; Yağız, 1999, p. 73).

Ertas also includes “poverty” 13 times in his poems. He states that the rich are much more valued in society and that the poor are not cared for and are despised:

*Oh, my lonely, suffering friend!*

*Why is not your spring or winter shadowless?*

*Do you have—they do not ask—bread to eat?*

*If you are rich, they say either Bey or Pasha*

*If you are poor, they say either Abdal or Cingan* (Gypsy)*!*

(Ey garip gönüllüm, dertli yoldaşım

Niye belli değil baharın, kışın?

Var mıdır, sormazlar; ekmeğin, aşın

Zengin isen; ya Bey derler, ya Paşa

Fakir isen; ya Abdal derler, ya Cingan hâşâ!; Parlak, 2013, p. 389).

 

 

*They do not say, “Who asks about his condition?”*

*They do not say, “He is low in the eyes of the ignorant.”*

*They do not say, “Who gives jobs to the lonely?”*

*If you are rich, they say either Bey or Pasha*

*If you are poor, they say either Abdal or Cingan* (Gypsy)*!*

(Kim onun halını sormuş, demezler

Cahilin gözünde hormuş, demezler

Gariplere kim iş vermiş, demezler

Zengin isen; ya Bey derler, ya Paşa

Fakir isen; ya Abdal derler, ya Cingan hâşâ!; Parlak, 2013, p. 389).

#### 3.1.3. Love

To love and be loved may be among the highest feelings one can experience in the world. However, when love is one-sided, the lover can suffer. Thereby, people falling in love are likely to confront all kinds of difficulties.

Mahzuni includes “love” 20 times in his poems. According to him, all kinds of troubles come to those falling in love:

*I can no longer trust the tombstone*

*The act of God is mysterious*

*Do you know what happens to lovers?*

*If you are afraid, do not be Mahzunî again!*

(Artık güvenemem mezar taşına

Akıl ermez şu Hüda’nın işine

Neler gelir Asikların başına

Korkarsan Mahzunî olma bir daha; Yağız, 1999, p. 67).

In another poem, he expresses suffering because of his love for a beautiful woman:

*It is incomprehensible why God did this!*

*Do you know what happens to the braves?*

*I am burned of her moon face, crescent brow*

*Ah! My life is running out.*

(Akıl ermez oldu neden işine,

Neler gelir yiğitlerin başına

Yandım mah (ay) yüzüne hilâl kaşına

Ah çektikçe ömrüm sökülmektedir; Yağız, 1999, p. 123).

In Ertas’s poems, the most frequently used concept that evokes suffering is “love.” He uses it 91 times in his poems and states that when love falls into one’s heart, it burns and ruins it.

*Flames of love captured my lonely heart*

*This heart of mine is on fire, dear for you!*

*The arrow of suffering sank into my lonely heart*

*This heart of mine is bleeding, dear for you!*

(Aşk ataşı düştü garip gönlüme

Yanıyor bu. gönlüm, yar senin için

Derdin oku battı garip gönlüme

Kanıyor bu. gönlüm yar senin için; Parlak, 2013, p. 323).

Ertas says that love blows the lover’s mind and that they will almost burn to ashes when falling in love:

*Your love drove me crazy*

*Burned and turned me into ashes*

*Made me a slave to others*

*Oh dear, me!*

(Aşkın beni del’eyledi

Yaktı, yaktı, kül eyledi

El âleme kul eyledi

Yar beni, beni beni; Parlak, 2013, p. 325).

#### 3.1.4. Ignorance

Ignorance draws attention as a suffering-themed concept in both poets’ works. While Mahzuni says eight times in his poems that ignorance hurts people, Ertas uses it 10 times in his poems.

Mahzuni describes the impact of ignorance thus:

*Ignorance blew my mind*

*And made me feel cheap*

*We have no place in the world*

*Hear us, father!*

(Cehalet aklım uçurdu

Beni yerlere geçirdi

Yerimiz yoktur dünyada

Duy baba duy duy; Yağız, 1999, p. 64).

In one of his poems, Ertas emphasizes that ignorance is the source of all kinds of troubles and almost dehumanizes people:

*Because of illiteracy*

*Ignorance comes out and spreads!*

*Because of lovelessness and disrespect*

*One gets tired of humanity!*

(İlimsizlik, bilgisizlik yüzünden

Cehalet hortlayıp çıkar mı çıkar!

Sevgisizlik, saygısızlık yüzünden

İnsan, insanlıktan bıkar mı bıkar!; Parlak, 2013, p. 435).

#### 3.1.5. Longing

Referring to the desire to see something, a person, or a place, longing is intertwined with suffering in folk poetry. A longing person suffers because of a state of absence. While Mahzuni mentions “longing” seven times in his poems, it appears 65 times in Ertas’s poems.

Mahzuni expresses his longing for his village as follows:

*Oh! I’ve pined for seeing you, Bercenek* (Mahzuni’s village)

*Oh! Foggy, foggy our lands*

*My tears have become full of ash*

*Oh! Foggy, foggy our lands*

*If I cry for them, they think I’m crazy*

(Vay göresim geldi Berçenek seni

Dumanlı dumanlı oy bizim eller

Aktı gözüm yaşı oldu bir çanak

Dumanlı dumanlı oy bizim eller

Oturup ağlarsam delidir derler; Yağız, 1999, p. 64).

Ertas also states in one of his poems that longing for his loved one hurts him:

*Heart’s suffering, suffering for love*

*Longing is tough*

*One not suffering cannot know it*

*Ask the meaning of suffering to sufferers*

(Gönül derdi, yar derdi

Hasret; yaman, zor deddi

Onu çekmeyen bilmez

Çekenlere sor, derdi; Parlak, 2013, p. 427).

#### 3.1.6. Death

Acknowledged to be as natural in human life as birth, death does not bring happiness to people as much as birth. Since death is conceived of as a kind of separation, poets consider it to be among those concepts that bring suffering. While “death” is mentioned three times in the poems by Mahzuni, it appears 10 times in Ertas’s poems.

Mahzuni states that many people die and are sent to cemeteries every year, which makes one suffer in this world:

*Oh, the cemetery full of flesh*

*Those coming to you never end*

*Damn, big world!*

*Those smashing each other never end*

(Yürü bre bol mezarlık

Her yılda varan varana

Kahrolasın koca dünya

Birbirin kıran kırana; Yağız, 1999, p. 96).

Ertas also conveys his longing for his father in his poem dedicated to his father:

*I’ve come a long way, for my longing*

*Where is my father, Muharrem?*

*Why does my wounded nightingale not make a sound?*

*Oh, my afflicted father; where is that Kerem* (kindness)*?*

(Uzak yoldan geldim, hasretim için

Hani, nerde babam, Muharrem nerde

Yaralı bülbülüm ses vermez, niçin?

Yüreği yanığım, o Kerem nerde?; Parlak, 2013, p. 489).

#### 3.1.7. Slavery

Slavery also makes one suffer. While Mahzuni uses “slavery” five times in his poems, Ertas refers to this concept nine times in his works.

Stating that people have been suffering from slavery since the start of human existence, Mahzuni emphasizes his rebellion against this situation as follows:

*Are you descended from Adam or Noah?*

*My arm, where did you get this chain?*

*I was a man too in this world of the cruel*

*My arm, where did you get this chain?*

(Adem’den mi geldin Nuh’tan mı kaldın?

Kolum nerden aldın sen bu. zinciri

Ben de bir adamdım kahpe dünyada

Kolum nerden aldın sen bu. zinciri?; Yağız, 1999, p. 93).

Ertas also states that he lost his job, friends, and freedom when imprisoned:

*I outstayed my welcome in prisons*

*I lost my love, my friend*

*Are all my buddies mad at me?*

*Oh, prison! You burned me*

*I wish I got rid of the guard’s words*

(Hapishanelere attım postumu

Kayıp ettim yârenimi, dostumu

Bütün ahbaplarım bana küstü mü?

Yandım mahpushane, senin elinden

Kurtulaydım gardiyanın dilinden; Parlak, 2013, p. 489).

#### 3.1.8. Injustice

Injustice is among the conditions that hurt people. While one can identify it in four places in Mahzuni’s verses, Ertas does not use it in his works.

Mahzuni emphasizes that people are likely to suffer in the absence of a conscientious distinction between right and wrong:

*Unless justice from the conscience*

*Unless the unjust is seen in the way of the Lord*

*Unless a deputy gets tired like a peasant*

*Neither bandit nor shepherd withers*

(Vicdan adaleti kurulmadıkça

Haksız hak yolunda görülmedikçe

Mebus köylü gibi yorulmadıkça

Ne eşkıya biter, ne çoban biter; Yağız, 1999, p. 65).

#### 3.1.9. Separation

Finally, this study addressed separation as a feeling that causes people to suffer. Despite its necessity from time to time, separation often brings sorrow to people. While it is not included in Mahzuni’s works, it appears 21 times in Ertas’s poems.

Ertas states that separation is sorrowful and that he experienced it many times:

*The pain of separation*

*Have I suffered less (x2)?*

*Its smoldering fire*

*Have I suffered less (x2)?*

(Ayrılığın acısını

Az mi çektim, az mı çektim

İçten içe sızışını

Az mı çektim, az mı çektim; Parlak, 2013, p. 328).

### 3.2. Happiness in the poems of Mahzuni and Ertas

The findings revealed that in both poets’ works, expressions implying happiness are used less often than those evoking suffering. In the poems analyzed, the researcher identified six themes implying happiness: love, friend, mother, soft answer, spring, and misery/remedy. While these concepts are repeated seven times in Asik Mahzuni Serif’s poems, they are found 132 times in Ertas’s works.

#### 3.2.1. Love

Love may be considered the most prominent source of happiness for people. While it is mentioned only two times in Mahzuni’s poems, it is included 89 times in Ertas’s works, the most frequently cited happiness-themed concept.

In one of his poems, Mahzuni says that life will be much more appealing when people love each other:

*When two hearts become one*

*It will be a feast, a relief*

*When two hearts engage in one joy*

*It will be a feast, a relief*

*One sacrifices himself for his friend*

(İki gönül bir olunca

Bayram olur seyran olur

İki gönül bir sevince

Bayram olur seyran olur

Bir dost dosta kurban olur; Yağız, 1999, p. 161).

Ertas describes how love makes him happy and how it causes changes to his body and soul:

*I’ve crossed the garden wall*

*And knotted into ivy roses*

*I’ve kissed, loved, and said goodbye to my lover*

*Oh! I’m burning (x3)*

*I’m fallen into the rosebud*

*Persian shawl to her wasp waist*

(Bahçe duvarından aştım

Sarmasik güllere dolaştım

Öptüm, sevdim, helalleştim

Yanıyorum, yanıyorum, yanıyorum hele

Mayil oldum gonca güle

Acem şalı, ince bele; Parlak, 2013, p. 334).

#### 3.2.2. Friend

Friendship occupies a substantial place in life since humans are social beings. Having a reliable friend brings happiness and confidence. While mentioned 17 times in Ertas’s poems, this theme is not encountered in Mahzuni’s works.

Ertas states that having a friend brings happiness and removes worries and sorrows:

*When sending regards to friends*

*When friends receive my greetings*

*When the lover smiles at me*

*Will there be suffering on Job’s skin, for God’s sake, hey!*

(Dostlara selamı saldıktan keri

Dostlar selamımız aldıktan keri

Canan yüzümüze güldükten keri

Dert kalır mı Eyüp teninde, ya hu, dost, ya hü!; Parlak, 2013, p. 384).

#### 3.2.3. Mother

The fact that maternal affection is often cited in the works of Turkish literature may indicate its significance. Maternal affection is known to be a source of happiness. While “mother” is mentioned 14 times in Ertas’s poems, it is not mentioned in Mahzuni’s works.

Ertas emphasizes that one’s mother is a great source of happiness and that she shines like the Sun:

*You’re a match for hearts*

*You’re a love burning in hearts*

*You’re the Sun to hearts*

*You’re divine light, Mother (x2)*

(Her gönüle bir eşsin sen

Kalpte yanan bir aşksın sen

Gönlümüze güneşsin sen

Nursun ana, nursun ana; Parlak, 2013, p. 361).

#### 3.2.4. Soft answer

Fruitful human relations are attributed to kindness, delicacy, and grace. Many cultures even have a proverb saying, “A soft answer turns away wrath.” People responding politely and elegantly may have an influence over others, which becomes a source of happiness for everyone engaged in such conversations. While “soft answer” is mentioned eight times in Ertas’s poems, Mahzuni does not refer to it in the poems analyzed.

Ertas states that a soft answer will sweeten the mouth like honey; that is, the kind and elegant speech will have a positive and pleasing effect on people:

*A thousand and one tastes for tongues*

*All for humans*

*They say sweet, for honey*

*Nothing is sweeter than the lover*

(Bin bir tat var. diller için

Hepsi de kullar için

Tatlı derler, ballar için

Yardan tatlısı bulunmaz; Parlak, 2013, p. 488).

#### 3.2.5. Spring

Spring is the season of revival referring to happiness. The blooming of flowers, the greening of trees, the warming of the weather, and the waking of animals from their hibernation in spring often evoke the joys of life. While “spring” is included four times in Ertas’s poems, it is not mentioned in Mahzuni’s works.

Ertas notes that flowers bloom and rose scents spread when spring comes. He regards it as a season that makes people happy:

*Spring has arrived; all kinds of flowers have bloomed*

*The rose in spring; how beautiful the rose is in spring*

*Buds have bloomed, roses are sprinkled*

*The rose in spring; how beautiful the rose is in spring*

(Bahar gelmiş, türlü çiçek açılmış

Baharda gül, gül, baharda ne güzel

Açılmış goncalar, güller saçılmış

Baharda gül, gül, baharda ne güzel; Parlak, 2013, p. 332).

#### 3.2.6. Misery/remedy

If an individual learns lessons from adverse conditions and grows as a person, they will be able to cope with subsequent adverse events. Thus, people with robust traits try to turn something negative in their favor by learning from past experiences. While “misery/remedy” is mentioned five times in Mahzuni’s works, it could not be found in Ertas’s poems.

Mahzuni emphasizes that people can sometimes learn from their miseries and use them to “cure” themselves:

*Mahzuni Serif, kill your pain!*

*Sometimes, find your remedy in misery*

*Like Pir Sultans, the gallows*

*I do not know, should I get hanged on or not?*

(Mahzuni Serif’im dindir acını

Bazen acılardan al ilacını

Pir Sultanlar gibi darağacını

Bilmem boylasam mı, boylamasam mı?; Yağız, 1999, p. 73).

## 4. Discussion

Recent developments in positive psychology have evolved into the second and third waves, going beyond the individual and positive focus toward multi-cultures, complex systems, and the psychology of transcending suffering (Wong et al., 2022). Second-wave positive psychology (PP 2.0) has, especially, emphasized the importance of suffering for growth and wellbeing (Wong et al., 2021) and of understanding the expression of happiness in the cultural context (Wong, 2011). In addition, PP 2.0 focuses on suffering as the gateway to happiness (Wong, 2019) and underlines that happiness is impossible without overcoming the dark side of human life (Wong, 2020). Therefore, it is important to examine suffering and happiness in the cultural context to provide a better understanding of the association between these constructs. To this end, the present study aims to provide a further understanding of suffering and happiness in Turkish folk culture through a sample of poems by Asik Mahzuni Serif and Neset Ertas.

Poetry is a form of art that helps people to express feelings, thoughts, and suffering, which may also be an effective intervention for improving mental health and wellbeing (McArdle and Byrt, 2001; Tegnér et al., 2009). Although the mechanism by which happiness works remains elusive, cultures are known to exert a substantial influence on the attributions of happiness. Given that culture is an individual’s lifestyle in the simplest sense (Taylor, 1871), folk poetry may be among its integral features. Findings from this study have supported this notion, indicating that suffering is *the gateway to happiness* (Wong, 2019). Suffering is mentioned more than happiness in the poems. Consistent with the second-wave positive psychology approach (Wong, 2011, 2019), the results have suggested that suffering is essential to establish flourishing, and people can make a better life by accepting and overcoming suffering.

While suffering was mentioned 400 times in the poems, happiness appeared 139 times. The concepts clustered under the theme of suffering were identified as the world, poverty, love, ignorance, longing, slavery, injustice, and separation. Of these, the world was cited most often in the works of Mahzuni (f: 35), although Ertas also used it extensively (f: 75). Both poets used the concept of the world in a social sense and state that the world is a painful place for people. Poverty was used more extensively in Mahzuni’s poems (f: 24) than in Ertas’s (f: 13). The same applied to injustice (f: 4 and 0, respectively). Although it might be thought that Mahzuni tends toward more social issues, similar frequencies of the use of ignorance (f: 8 and 10, respectively) and slavery (f: 5 and 9, respectively) in both poets’ works imply that Ertas is not insensitive to social issues either. The poems also included the concepts of longing and death in relation to suffering. Finally, the concept of separation, which was not mentioned in Mahzuni’s works but used 21 times in Ertas’s poems, can also be considered an element leading to individual suffering. These results suggest that, in Turkish folk poetry, poets often reflect social troubles, sorrows, and happiness. While sometimes expressing misery in regard to disasters and adverse social events where people are harmed, folk poets also sometimes convey love through the arrival of spring and happiness in their poems.

Previous research on Ertas’s works (Akgün, 2006; Parlak, 2013; Aktaş and Şimşek, 2014) has consistently reported that he is a poet of suffering and misery; it has always mentioned the cries of suffering in his poems called “Bozlak.” Mahzuni also mainly addresses societal problems, troubles, and suffering. For this reason, it can be concluded that the concepts of ignorance, slavery, injustice, and poverty are more evident in both poets’ works in relation to the theme of suffering, a finding that overlaps with the views of Yağız (1999) and Parlak (2013). However, it is interesting that Mahzuni rarely adopted happiness-oriented concepts in his works. While he used such images only seven times in his poems, they appear 132 in Ertas’s works. The most frequently used happiness-oriented concept in Ertas’s works was determined to be love (f: 89), but only two poems of Mahzuni included this concept. Moreover, the concepts of the friend (f: 17), mother (f: 14), soft answer (f: 8), and spring (f: 4), which appeared in Ertas’s works, could not be found at all in Mahzuni’s poems. It can be concluded that Ertas adopted these concepts in relation to the poet himself. In addition, the concept of misery/remedy was identified in the poems of Mahzuni but not in those of Ertas. From the perspective of positive psychology, Mahzuni emphasizes that suffering is a cause of upsets but also an important source of building resilience, which, in turn, can produce happiness. In other words, an individual can grow with the help of the experience of suffering so that that misery can become a remedy.

In summary, both poets extensively used the suffering theme in their poems and paid less attention to the happiness theme. This may be due to both poets’ adverse experiences during their lifetime. Considering the Turkish cultural context, suffering is also a key source of growth, resilience, and flourishing for human beings. According to both poets, life is short and temporary. In the world, we constantly witness all kinds of injustice, and we suffer because of what we go through. From this point of view, although the world seems not to be a place of happiness for either poet, we should persist in life with an attitude of love, consider negatives as an opportunity to grow, and try to realize ourselves since the negatives in this short life are all temporary.

## 5. Conclusion and limitations

The present study aimed to explore suffering and happiness in the poems of two prominent representatives of Turkish folk poetry in the 21st century, Asik Mahzuni Serif and Neset Ertas, in the context of second-wave positive psychology. The study results have first revealed that suffering is an inescapable part of human life and is widely utilized in Turkish folk poetry. Then, suffering is a cause of upsets but also an important source of building resilience, which, in turn, can produce happiness. These findings suggest that suffering makes people stronger and better, and the best way to achieve durable happiness is to overcome suffering. However, further research needs to provide a deep understanding of the relationship between suffering and happiness in the cultural context. For instance, folk poetry as a therapeutic intervention may improve resilience and happiness, which, in turn, reduces suffering; however, randomized control studies are needed.

The results of this study should also be considered in light of some limitations. First, the study is limited to the data obtained from the poems of Mahzuni and Ertas. Therefore, future studies could be examined the poems by different poets in the Turkish culture to provide a deep understanding of the link between poems and positive psychology. Furthermore, research might be provided comparisons with poets from other countries, other classes, and genders. Next, both poets are considered contemporary folk poets; therefore, further research can be recommended to explore the poems by classical folk poets and to compare the results with those obtained in this research. In this way, it may be convenient to identify the themes that dominate particular centuries and to discuss the period-specific conditions.

## Data availability statement

The original contributions presented in the study are included in the article/supplementary material, further inquiries can be directed to the corresponding author.

## Author contributions

AG and GA contributed to the design of the study. AG wrote the introduction, results, and discussion sections. GA edited and improved the manuscript. All authors contributed to the article and approved the submitted version.

## Conflict of interest

The authors declare that the research was conducted in the absence of any commercial or financial relationships that could be construed as a potential conflict of interest.

The handling editor PW declared a past co-authorship with the author GA.

## Publisher’s note

All claims expressed in this article are solely those of the authors and do not necessarily represent those of their affiliated organizations, or those of the publisher, the editors and the reviewers. Any product that may be evaluated in this article, or claim that may be made by its manufacturer, is not guaranteed or endorsed by the publisher.
